# E-cadherin: A determinant molecule associated with ovarian cancer progression, dissemination and aggressiveness

**DOI:** 10.1371/journal.pone.0184439

**Published:** 2017-09-21

**Authors:** Marina Rosso, Blanca Majem, Laura Devis, Lara Lapyckyj, María José Besso, Marta Llauradó, María Florencia Abascal, María Laura Matos, Lucia Lanau, Josep Castellví, José Luis Sánchez, Asunción Pérez Benavente, Antonio Gil-Moreno, Jaume Reventós, Anna Santamaria Margalef, Marina Rigau, Mónica Hebe Vazquez-Levin

**Affiliations:** 1 Laboratorio de Estudios de la Interacción Celular en Reproducción y Cáncer, Instituto de Biología y Medicina Experimental (IBYME; CONICET-FIBYME), Buenos Aires, Argentina; 2 Biomedical Research Unit in Gynecology, Vall Hebron Research Institute and University Hospital, Barcelona, Spain; 3 Pathology Department, Vall Hebron University Hospital, Barcelona, Spain; 4 Gynecology Oncology Department, Vall Hebron University Hospital, Barcelona, Spain; University of Navarra, SPAIN

## Abstract

Ovarian cancer (OC) is the fifth cancer death cause in women worldwide. The malignant nature of this disease stems from its unique dissemination pattern. Epithelial-to-mesenchymal transition (EMT) has been reported in OC and downregulation of Epithelial cadherin (E-cadherin) is a hallmark of this process. However, findings on the relationship between E-cadherin levels and OC progression, dissemination and aggressiveness are controversial. In this study, the evaluation of E-cadherin expression in an OC tissue microarray revealed its prognostic value to discriminate between advanced- and early-stage tumors, as well as serous tumors from other histologies. Moreover, E-cadherin, Neural cadherin (N-cadherin), cytokeratins and vimentin expression was assessed in TOV-112, SKOV-3, OAW-42 and OV-90 OC cell lines grown in monolayers and under anchorage-independent conditions to mimic ovarian tumor cell dissemination, and results were associated with cell aggressiveness. According to these EMT-related markers, cell lines were classified as mesenchymal (M; TOV-112), intermediate mesenchymal (IM; SKOV-3), intermediate epithelial (IE; OAW-42) and epithelial (E; OV-90). M- and IM-cells depicted the highest migration capacity when grown in monolayers, and aggregates derived from M- and IM-cell lines showed lower cell death, higher adhesion to extracellular matrices and higher invasion capacity than E- and IE-aggregates. The analysis of E-cadherin, N-cadherin, cytokeratin 19 and vimentin mRNA levels in 20 advanced-stage high-grade serous human OC ascites showed an IM phenotype in all cases, characterized by higher proportions of N- to E-cadherin and vimentin to cytokeratin 19. In particular, higher E-cadherin mRNA levels were associated with cancer antigen 125 levels more than 500 U/mL and platinum-free intervals less than 6 months. Altogether, E-cadherin expression levels were found relevant for the assessment of OC progression and aggressiveness.

## Introduction

Ovarian cancer (OC) is the seventh most common cancer and the fifth cause of cancer death in women worldwide [1]. Epithelial OC is the most frequent type, comprising 90% of all cases [2]. Largely asymptomatic, more than 70% of patients affected with this disease are diagnosed at an advanced stage, with a 5-year survival rate lower than 20% [3].

The malignant nature of OC stems from its unique dissemination pattern and consequent metastatic behavior; tumor cells can spread directly throughout the peritoneal cavity due to the lack of an anatomical barrier. OC peritoneal metastasis relies on the ability of exfoliated primary tumor cells to aggregate in multicellular structures, survive in suspension and subsequently adhere to and infiltrate the mesothelial lining of the peritoneum and omentum [3]. This “seeding” of the abdominal cavity is also associated with ascites formation (accumulation of malignant fluid) and is responsible for most of the OC morbidity and mortality [4].

In solid tumors, the loss of cellular contacts contributes to distortion of normal tissue architecture and promotes cancer progression and dissemination. Among proteins involved in epithelial cell-cell adhesion, Epithelial cadherin (E-cadherin) plays a key role. E-cadherin is the founder member of the cadherin superfamily, a group of cell surface glycoproteins that mediate calcium-dependent cellular adhesion [5]. The human E-cadherin gene, called *CDH1*, encodes a 120 kDa mature single-span transmembrane protein localized at the plasma membrane of epithelial cells. Whereas the E-cadherin extracellular domain is involved in cellular adhesion, the intracellular domain interacts with the actin cytoskeleton to strengthen cell-cell interactions by means of adaptor proteins, i.e. β-catenin, and participates in signal transduction pathways [6].

E-cadherin has been defined as a tumor suppressor, since it has been frequently found downregulated in malignant epithelial tumors [7–9]. Several mechanisms have been involved in E-cadherin deregulation, among them loss of heterozygosity at the 16q22.1 chromosome region, occurrence of *CDH1* inactivating mutations, *CDH1* gene promoter hypermethylation, overexpression of E-cadherin transcriptional repressor factors and post-translational modifications (i.e. phosphorylation and glycosylation) [10]. Associated to the decrease in E-cadherin levels, epithelial cells may acquire a mesenchymal phenotype, losing cell-cell adhesion and gaining a more motile and invasive behavior [11]. This process is known as epithelial-to-mesenchymal transition (EMT) and has been recognized as a key event not only during embryonic development, but also under pathological conditions such as cancer progression [12].

Cellular changes characteristic of the EMT process occur in association with protein and gene expression modifications, among them reduced levels of epithelial intermediate filament-forming proteins (i.e. cytokeratins), overexpression of type III mesenchymal intermediate filament protein, called vimentin, and alterations in cell-cell and cell-matrix adhesion molecules [13]. Another key feature of the EMT process is the “cadherin switch” phenomenon, in which E-cadherin downregulation is associated with Neural cadherin (N-cadherin) expression [14]. This switch has been related with an increased cell motility and cell invasion capacity [14, 15], and it can be regulated by a number of zinc-finger transcription factors that negatively modulate E-cadherin expression, including Twist, Snail, Slug, ZEB1, among others [16, 17]. Moreover, the ability to overcome anoikis, a programmed cell death induced upon cell detachment from the extracellular matrix (ECM), is also associated with the acquisition of a mesenchymal phenotype and confers an invasive cellular behavior [18].

Although changes in E-cadherin and other EMT-related markers have been reported in OC, information on the relationship between their expression levels and tumor progression, dissemination and aggressiveness is still limited and controversial [19, 20]. To address these issues, the following studies were carried out: i) the expression and sub-cellular localization of E-cadherin was characterized in an OC tissue microarray (TMA) by immunohistochemistry, and results were associated with a set of clinicopathological parameters; ii) a molecular expression analysis of E-cadherin and EMT-related markers was done in 4 OC cell lines grown in monolayers and under anchorage-independent conditions to mimic OC dissemination; iii) a functional characterization was done in the 4 OC cell lines grown under anchorage-independent conditions by evaluating cell death, adhesion, migration and invasion properties; iv) a quantification analysis of E-cadherin and EMT-related markers mRNA expression levels was done in tumor- and ascites-primary cultures derived from patients with advanced-stage high-grade serous OC, and results were associated with disease aggressiveness and patient prognosis.

## Materials and methods

### Materials

#### Chemicals

Chemicals were of analytical or tissue culture grade and purchased from Sigma-Aldrich (Sigma; St. Louis, MO, USA). Molecular biology reagents were purchased from Invitrogen-Life Technologies (Carlsbad, CA, USA) and Qiagen (Hilden, Germany). Electrophoresis reagents were products of BioRad (Richmond, CA, USA). The following antibodies were used: anti E-cadherin a) 610181 (mouse, monoclonal; Becton Dickinson Biosciences [BD], San Diego, CA, USA) and b) H-108 (rabbit, polyclonal; Santa Cruz Biotechnology [SCBT], Santa Cruz, CA, USA); anti N-cadherin a) H63 (rabbit, polyclonal; SCBT) and b) 610920 (mouse, monoclonal; BD); anti β-catenin (E247; rabbit, monoclonal; Abcam, Cambridge, UK); anti pan-cytokeratin (AE1/AE3; mouse, monoclonal; SCBT); anti poly-(ADP-ribose) polymerase-1 (PARP-1) (H250; rabbit, polyclonal; SCBT); anti paxillin (610619; mouse, monoclonal; BD); anti vimentin (clone V9; mouse, monoclonal; Dako, Glostrup, Denmark); anti actin (A2668; rabbit, polyclonal; Sigma); and anti β-tubulin (clone D66; mouse, monoclonal; Sigma). For immunocytochemistry protocols, Alexa Fluor 488 or 555 goat-labeled anti-mouse and anti-rabbit immunoglobulins G were used as secondary antibodies. Anti-mouse (Vector Lab. Inc., Burlingame, CA, USA) or anti-rabbit (Sigma) immunoglobulins G coupled to horseradish peroxidase were employed as secondary antibodies in Western immunoblotting assays.

#### Patient samples

Tissue samples and ascites were obtained at the operating room from OC patients who underwent surgery before receiving hormonal and/or chemotherapy treatment at the Department of Gynecological Oncology of Vall Hebron Hospital, Barcelona, Spain. The Institutional Review Board approved the protocol and a written informed consent was obtained from all patients participating in the study. Clinical data was obtained from the Gynecological Oncology database of the Department of Gynecological Oncology of Vall Hebron Hospital. Cancer antigen 125 (CA125) levels (U/mL) were determined at the time of diagnosis, prior to neither surgery nor chemotherapy. Platinum-free interval (PFI) was measured as the disease-free period (months) after the end of chemotherapy. All patients received the same chemotherapy based on 6 cycles of a combination of paclitaxel and carboplatin drugs.

Studies were performed with both formalin-fixed paraffin-embedded (FFPE) and fresh OC samples. FFPE samples retrieved from the Pathology Department of Vall Hebron Hospital were collected between 1999 and 2008, and were used to construct a TMA, as previously described [21]. Representative areas of 76 ovarian carcinomas (32 serous [42.1%], 13 mucinous [17.1%], 14 endometrioid [18.4%], 11 clear cell [14.5%] and 6 undifferentiated [7.9%]) were included. From the 76 FFPE tumor-tissue samples, 27 (35.5%) and 49 (64.5%) were graded as low and high grade, respectively, based on tumor cell differentiation. Considering the International Federation of Gynecology and Obstetrics (FIGO) staging system, 48.7% of tumors included in the TMA belonged to early stages (Stage I: 27 and Stage II: 10), while 51.3% were classified as advanced stages (Stage III: 36 and Stage IV: 3). Moreover, a total of 6 fresh tumor-tissue samples and 20 ascites derived from patients with advanced-stage high-grade serous OC were collected at the Department of Gynecological Oncology of Vall Hebron Hospital between 2012 and 2014, and were processed to develop primary cell cultures.

For the *in silico* analysis, gene expression data generated by Agilent array technology was obtained from the Ovarian Serous Cystadenocarcinoma database available at The Cancer Genome Atlas (TCGA) data portal (https://cancergenome.nih.gov/). Sample data was downloaded from the UCSC Xena website (AgilentG4502A_07_3; https://xenabrowser.net/datapages/?dataset=TCGA.OV.sampleMap/AgilentG4502A_07_3&host=https://tcga.xenahubs.net; October 2016), processed, and selected output information was analyzed and presented in this report.

Information of *CDH1* somatic mutations identified in human epithelial ovarian tumors of serous histology was retrieved from the Catalog Of Somatic Mutations In Cancer (COSMIC) website (http://cancer.sanger.ac.uk/cosmic/browse/tissue-sn=ovary&ss=all&hn=all&sh=serouscarcinoma&in=t&src=tissue&all_data=n; October 2016) and summarized in this report.

#### Cell lines

The TOV-112, SKOV-3, OAW-42 and OV-90 human OC cell lines (American Type Culture Collection, Manassas, VA, USA) **(Table 1)** were selected to carry out standard monolayer and anchorage-independent cell cultures.

**Table 1:** General characteristics of OC cell lines.

*OC Cell Line*	*Tumor Type*	*Source*	*Culture Media*
TOV-112	Ovarian Endometrioid Adenocarcinoma–High Grade, Stage IIIC	Primary tumor	MCDB105:M199 (1:1); supplemented with 10% Fetal Bovine Serum and 1% Penicillin-Streptomycin
SKOV-3	Ovarian Adenocarcinoma	Ascites	McCoy's 5A; supplemented with 10% Fetal Bovine Serum, 1% Penicillin-Streptomycin, 0.1% Hepes and 0.7% Fungizone
OAW-42	Ovarian Adenocarcinoma	Ascites	DMEM High Glucose (4.5 g/L) with L-Glutamine; supplemented with 10% Fetal Bovine Serum and 1% Penicillin-Streptomycin
OV-90	Ovarian Serous Papillary Adenocarcinoma–High Grade, Stage IIIC	Ascites	MCDB105:M199 (1:1); supplemented with 15% Fetal Bovine Serum and 1% Penicillin-Streptomycin

### Methods

#### Monolayer cell cultures

OC cell lines were cultured at 37°C and 5% CO_2_ in air, following supplier´s instructions and appropriate culture conditions **(Table 1)**.

#### Primary cultures

For tissue-derived primary cultures, the surface of OC tumors was scraped off, cells were placed in culture medium and centrifuged for 5 minutes at 1200 rpm. The cell pellet was resuspended in 10% fetal bovine serum (FBS) MCDB105:M199 (Biological Industries, Kibbutz Beit-Haemek, Israel) (1:1) culture medium supplemented with 1% penicillin-streptomycin, and placed into a 6-well plate (Nunc-Thermo Scientific, Waltham, MA, USA). To reduce fibroblast contamination, non-attached cells (epithelial cells) were placed into a new well 30 minutes later. After 7 days, the medium was changed and cell morphology was monitored until 90% confluence, after which cells were frozen.

For ascites-primary cultures, the ascitic fluid was mixed 1:1 with MCDB105:M199 (1:1) culture medium supplemented with 10% FBS and 1% penicillin-streptomycin, placed in a T-75 flask (Nunc-Thermo Scientific) and incubated 7 days at 37°C and 5% CO_2_ in air. Then, the conditioned medium was removed to eliminate non-adherent cells (i.e. cells of the immune system and blood) and replaced by fresh medium; ascitic cells were then handled as tumor-primary cultures.

#### Anchorage-independent cell cultures

OC cell lines were grown under anchorage-independent conditions using 2 methodologies:

*Hanging drop method*. The procedure was done as previously described [22]. Briefly, adherent cell monolayers were harvested, and cell suspensions were counted and subsequently diluted to 1x10^5^ cells/mL. Drops of 2000 cells in 20 μL were plated in the lid of a p100 dish and cultured for up to 48 hours. Images were taken at 24 and 48 hours to evaluate cell morphology, and analyzed using the Image J software (Wright Cell Imaging Facility, UHNR, CA, USA). Forty-eight hour-aggregates were recovered for immunocytochemical and functional analyses.

*Liquid overlay method*. The assay was performed as previously reported [23]. Basically, 6-well plates were coated with 0.5% agarose (SeaKem LE agarose, Lonza, Basel, Switzerland) in FBS-free medium and left at 4°C during 30 minutes for solidification. A total of 8x10^5^ cells were seeded in each well. Forty-eight hour-aggregates were recovered for total RNA and protein analyses.

#### Immunohistochemistry

E-cadherin was detected by an indirect immunoperoxidase assay in 76 OC tumors arranged in a TMA, as earlier described. For antigen retrieval, a 2 minute-incubation at 115°C in 10 mM citrate buffer (pH = 7.3) was done. Sections were incubated with an anti E-cadherin antibody (610181; 5 μg/mL) for 1 hour at room temperature, followed by a 30 minute-incubation with peroxidase-conjugated rabbit anti-mouse immunoglobulin G (EnVision, Dako), and detected using the Envision Plus Detection System (Dako).

Two spots from different areas of each of the 76 OC tumors included in the TMA were selected and evaluated by 2 highly-experienced pathologists. The intensity of the E-cadherin protein signal was measured at the plasma membrane, cytoplasm and nucleus. Staining intensity scores were assigned to each tumor evaluated, by applying a numerical scale ranging from 0 to 3, where 0 was assigned to lack of staining and 1 to 3 values were given to increasing tumor staining intensities (1 lowest and 3 highest). The resulting scores were dichotomized to analyze their relationship with the corresponding clinicopathological parameters (FIGO staging, grade of differentiation and histology). The cut-off values were set from the statistical distributions and the sensitivity/specificity of the receiver operating characteristic (ROC) curves, in relation with their capability to differentiate clinicopathological variables, as follows: Positive>1 and Negative≤1 for the membranous and cytoplasmic subcellular localizations, and Positive>0 and Negative = 0 for the nuclear staining. The total E-cadherin score was calculated from the membranous, cytoplasmic and nuclear scores, and was considered positive when a positive result was obtained for at least one of the subcellular localizations.

#### Fluorescence immunocytochemistry

OC cell lines grown in monolayers and 48 hour-aggregates generated using the hanging drop method were fixed with 4% paraformaldehyde, permeabilized in 0.1% Triton X-100, blocked with 3% bovine serum albumin (BSA) and subjected to fluorescence immunocytochemistry. Briefly, fixed cell monolayers/aggregates were placed 1 hour with an anti E-cadherin (H-108; 4 μg/mL), β-catenin (4 μg/mL), N-cadherin (610920; 2.5 μg/mL) or paxillin (5 μg/mL) antibody, followed by one additional hour incubation with the secondary antibody. Nuclear cell staining was done with Hoechst 33342 (Sigma). Images were acquired with a Nikon laser confocal microscope C1 (Tokyo, Japan; excitation lines: 488 nm and 544 nm, emission filters: 515–530 nm and 570-LP nm); images were acquired using 20x and 40x objectives.

#### Sample preparation, SDS-PAGE and Western immunoblotting

Total protein cell lysates were obtained from OC cell lines grown in monolayers and under anchored-independent conditions, as previously reported [24]. Protein content was quantified (BioRad Protein Assay), and protein extracts were subjected to SDS-PAGE in 8–12% polyacrylamide gels and transferred onto nitrocellulose membranes (GE Healthcare, Buckinghamshire, UK). The immunodetection protocol was done as earlier reported [24]. Briefly, membranes were immersed in phosphate buffered saline (PBS) buffer containing 0.02% Tween-20 (v/v), and supplemented with 10% skim milk (w/v) for 1 hour at room temperature in order to block membrane non-specific sites. Membranes were then incubated overnight at 4°C in the presence of specific antibodies diluted in blocking buffer (anti E-cadherin 610181: 0.25 μg/mL; anti N-cadherin H-63: 2 μg/mL; anti vimentin: 2 μg/mL; anti pan-cytokeratin: 2 μg/mL; anti PARP-1: 1 μg/mL; anti actin: 0.27 μg/mL; and anti β-tubulin: 0.05 μg/mL). As secondary antibodies, anti-mouse or anti-rabbit immunoglobulins G coupled to horseradish peroxidase were diluted in blocking buffer (0.4 mg/mL) and incubated 1 hour at room temperature. The antibody binding was revealed with the ECL Western Blotting Detection Kit (GE Healthcare), following the manufacturer´s instructions. Replicates of 3 experiments were obtained and a densitometric analysis of the bands was performed using the Image J software, when indicated. A representative image of each experiment is shown.

#### RNA extraction, cDNA synthesis, standard and quantitative real time PCR

OC cell lines grown in monolayers and under anchored-independent conditions, as well as OC tumor- and ascites-primary cultures, were subjected to total RNA purification (All Prep DNA/RNA mini Kit, Qiagen). Then, total RNA was subjected to a reverse transcriptase reaction using the Superscript III enzyme (Invitrogen), and standard and quantitative PCR protocols were carried out as previously reported [24]. Transcript expression levels for all genes evaluated in this study were estimated by the 2^-ΔCt^ calculation, where ΔCt = (Ct gene under study–Ct endogenous gene). Glyceraldehyde 3-phosphate dehydrogenase (GAPDH) was considered as the housekeeping gene in all cases.

E-cadherin mRNA levels were similar when individual or pooled cell lines from 3 replicates were compared **(S1 Fig)**. Therefore, pooled samples were analyzed in the following experiments.

#### Wound healing assay

The wound healing assay was done with OC cell lines as previously reported [21]. Images were taken and analyzed using the Image J software. The wound area (wa; mm^2^) recorded at the initial time (wat0) and at 4, 8, 12, 24 and 48 hours (watx), were used to calculate the percentage (%) of wound healing as [(wat0-watx)/wat0]x100, where 100% is the maximum migratory rate.

#### Cell death analysis

Forty eight hour-aggregates were obtained by the hanging drop method. In each assay, 40 drops/cell line were collected, centrifuged, trypsinized to allow cell disaggregation and incubated 5 minutes with 5 μL of propidium iodide (PI) (BD). The total number of cells was counted using a phase contrast microscope and dead cells were scored under fluorescence microscopy. The percentage (%) of cell death was calculated as the ratio between PI-stained cells and the total number of cells.

#### Adhesion assay

Forty eight hour-aggregates from the 4 OC cell lines were generated using the hanging drop method. In each assay, 40 drops/cell line per condition were collected, seeded into fibronectin- and collagen I-coated coverslips, and allowed to adhere for 2 hours. Adhered aggregates were fixed, stained with crystal violet and photographed. The number of aggregates adhered to both ECM was manually quantified using the FSX100 microscope (Olympus, Tokyo, Japan) and the Image J software. At least 4 random fields per coverslip were evaluated for each cell line in each condition.

#### Disaggregation assay

The disaggregation assay was performed as previously described [25]. Briefly, 96-well plates coated with fibronectin and collagen I ECM were blocked with 1% BSA in PBS for 30 minutes. One 48 hour-cell aggregate generated using the hanging drop method was seeded per well. Aggregates were photographed using the FSX100 microscope (Olympus) at 1, 3, 6, 9, 24 and 30 hours. The pixel area of cell aggregates was manually calculated at each time using the Image J software. The fold change in the area was estimated dividing the aggregate pixel area at 3, 6, 9, 24 and 30 hours by the corresponding value at 1 hour, established as the initial time (t = 0). The average percentage increase in the surface area from at least 4 aggregates was calculated and plotted.

In addition, immunodetection of paxillin in 24 hour-cell aggregates interacting with fibronectin and collagen I ECM was performed as described above.

#### 3D-Matrigel™ invasion assay

Forty μL of Matrigel™ (BD) were pipetted into each well of a 96-well plate and incubated 30 minutes at 37°C. Five 48 hour-cell aggregates from each of the 4 OC cell lines generated by the hanging drop method were seeded per well and other 40 μL of Matrigel™ were added into each well. Cell aggregates were grown at 37°C and 5% CO_2_ in air, and photographed with a FSX100 microscope (Olympus) for 7 days.

#### CA125 and PFI assessments

CA125 serum levels were evaluated by a standard immunoassay protocol at the Department of Gynecological Oncology of Vall Hebron University Hospital, Barcelona, Spain [26]. The cut-off value selected for monitoring treatment response was 500 U/mL [27]. The PFI was set at 6 months, a time period established to distinguish chemotherapy responsive (more than 6 months) or resistant (less than 6 months) patients.

#### Statistical analysis

All experiments were run in triplicates. Results are expressed as mean ± standard error of the mean (SEM). A p<0.05 value was considered statistically significant in all cases. The one-way analysis of variance (ANOVA) and student *t* tests were applied to compare mean values of mRNA and/or protein expression levels, and the nonparametric Spearman’s rho test was used to analyze correlations in human samples.

The relationship between membranous, cytoplasmic, nuclear and total E-cadherin expression levels with clinicopathological parameters of the ovarian tumors included in the TMA was evaluated using the Fisher´s exact and chi-square tests. To analyze the potential value of membranous and total E-cadherin staining to differentiate clinicopathological parameters, a univariate logistic regression (LR) analysis was conducted and the odds ratio (OR) and confidence intervals established at 95% were calculated.

Statistical analyses were performed using the Statistical Package for Social Science software versions 16.0 and 21.0 (New York, NY, USA). For graphical images the GraphPad 5.0 software was used, and figures were performed with the Adobe Photoshop™ CS5 software.

## Results

### Expression of E-cadherin in an OC TMA and its relationship with clinicopathological parameters

**Fig 1:**
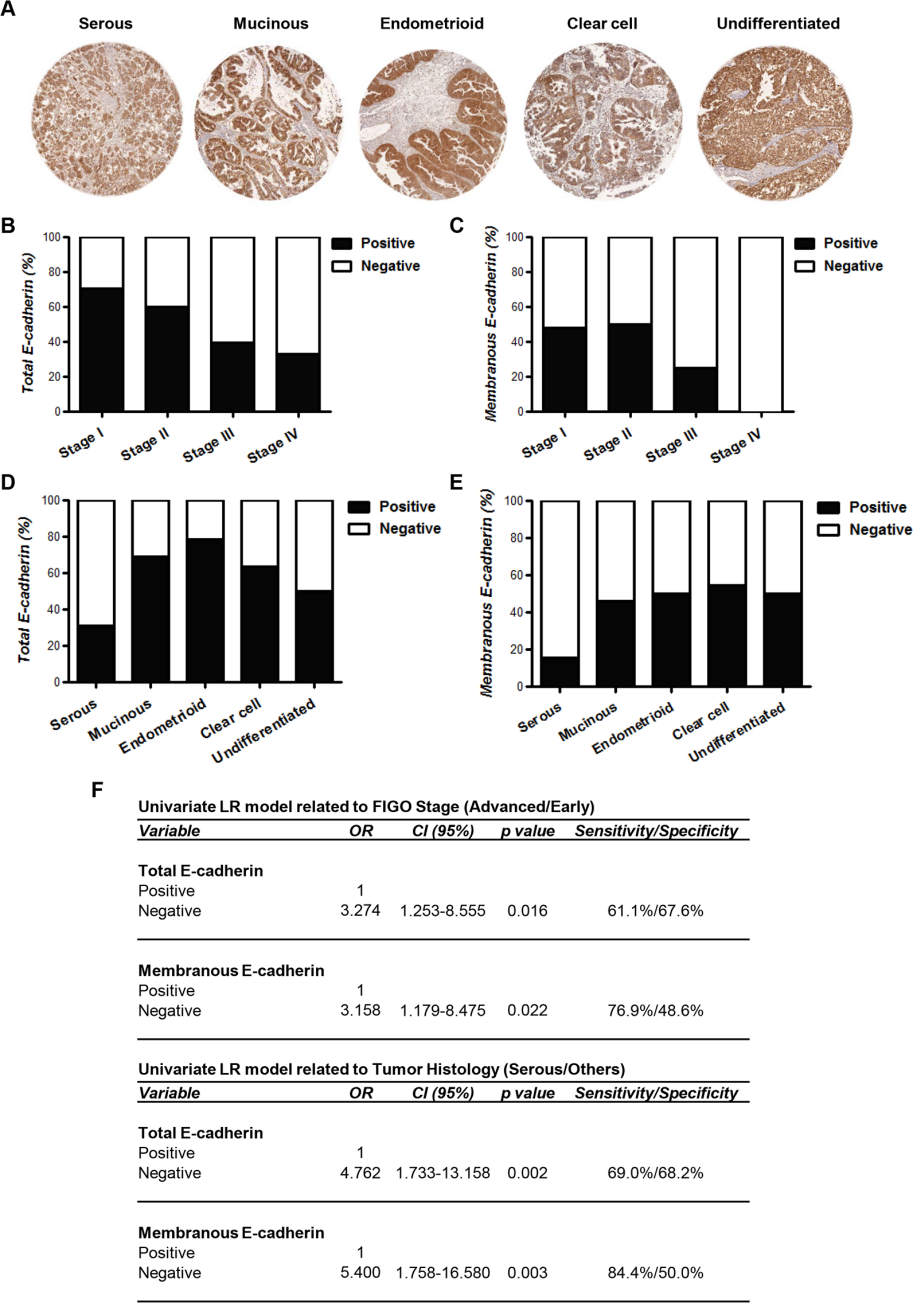
Immunohistochemical analysis of E-cadherin expression in a human OC TMA and its relationship with clinicopathological parameters. **(A)** E-cadherin immunodetection in a human OC TMA. Representative 100x magnification images are shown for high-grade tumors of different histological types. **(B and C)** Quantitative analysis (%) of **(B)** total and **(C)** membranous E-cadherin expression (Positive: black; Negative: white) in ovarian tumor tissues classified by FIGO stages. **(D and E)** Quantitative analysis (%) of **(D)** total and **(E)** membranous E-cadherin expression (Positive: black; Negative: white) in ovarian tumor tissues classified by histological types. **(F)** Univariate LR analysis of total and membranous E-cadherin expression levels versus FIGO stages (Advanced/Early Stages) and histological types (Serous/Others).

To study the relationship between E-cadherin and main clinicopathological parameters in ovarian tumor samples, the expression and subcellular localization of the adhesion protein was evaluated in an OC TMA. Representative images of E-cadherin staining are shown for high-grade ovarian tumors **(Fig 1A)**, as wells as for early- and advanced-stage tumors **(S2A Fig)**, of different histological types.

Among OC samples analyzed, 53.4% were positive for total E-cadherin, while 35.5% showed a membranous E-cadherin signal **(S2B and S2C Fig)**.

The analysis performed between E-cadherin expression and clinicopathological parameters revealed a significant decrease in total and membranous E-cadherin staining with increased FIGO stage (p = 0.017 and p = 0.027, respectively), reaching 66.7% and 100% negative staining in Stage IV tumors, respectively **(S2B and S2C Fig, Fig 1B and 1C)**. In line with these findings, advanced-stage tumors showed lower E-cadherin levels than early-stage tumors for both total and membranous protein (p = 0.019 and p = 0.030, respectively; **S2B and C Fig**). With regard to tumor histology, total E-cadherin expression varied significantly among different types (p = 0.023), being lowest in serous (31.0%) and highest in endometrioid (78.6%) tumors **(S2B Fig and Fig 1D)**. Similarly, for membranous E-cadherin significant differences (p = 0.028) were observed among histologies, finding lowest levels in serous tumors (15.6%) and highest levels in tumors with clear cell histology (54.5%) **(S2C Fig and Fig 1E)**. When the expression of the adhesion protein was analyzed with regard to tumor grade, no significant differences were found for total (p = 0.808) and membranous E-cadherin (p = 0.464) **(S2B and S2C Fig)**.

For the other cellular localizations, no significant differences were observed between cytoplasmic E-cadherin staining and tumor stage (p = 1.000), histology (p = 0.311) and grade (p = 1.000) **(S2C Fig)**. On the other hand, nuclear E-cadherin staining was found associated with tumor grade (p = 0.041), showing a higher signal in low-grade compared to high-grade tumors (26.9% versus 8.2%, **S2E Fig**).

To study the value of assessing total and membranous E-cadherin protein expression in the OC TMA, univariate LR analyses were performed. As a result, both negative membranous and total E-cadherin expression were validated as prognostic markers to discriminate advanced- from early-stage tumors, and serous histology among others. However, membranous E-cadherin was found more sensitive (76.9%) than total E-cadherin (61.1%) to identify advanced- from early-stage tumors, and to discriminate the serous histology among other histological subtypes (84.4% versus 69.0%, respectively). On the other hand, membranous E-cadherin was less specific than total E-cadherin staining for both FIGO stage (48.6% versus 67.6%) and tumor histology (50.0% versus 68.2%) **(Fig 1F)**.

### Evaluation of mechanisms involved in the regulation of E-cadherin expression levels in human serous ovarian tumors

Considering that serous ovarian tumors depicted the lowest levels of E-cadherin among tumor subtypes included in the TMA **(Fig 1D and 1E)**, the occurrence of somatic mutations and the expression of transcriptional E-cadherin repressor factors were evaluated in tumors of this histology.

Firstly, the COSMIC portal of somatic mutations in cancer was used to retrieve the mutations listed in the *CDH1* gene. Within the 737 serous ovarian tumors evaluated only 2 (0.27%) mutations were reported, being both substitution missense mutations that lead to changes in amino acid residues located in the E-cadherin extracellular domain.

**Fig 2:**
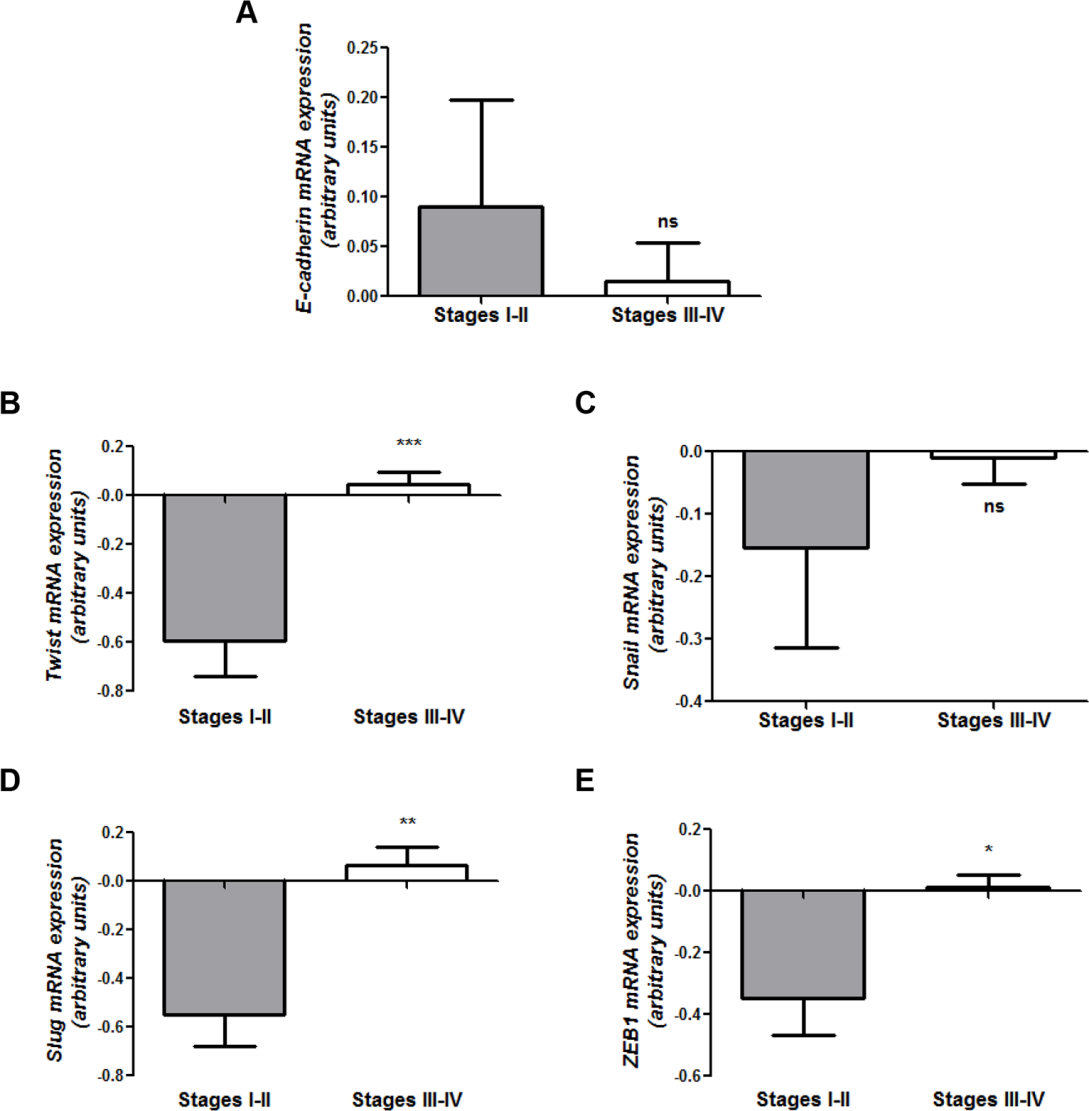
Expression analysis of E-cadherin, Twist, Snail, Slug and ZEB1 mRNA levels in early- and advanced-stage human serous ovarian tumors. **(A)** Quantitative real time PCR analysis of E-cadherin mRNA expression. GAPDH was used as endogenous control (ns: no significant). **(B-E)** Transcript expression levels of **(B)** Twist, **(C)** Snail, **(D)** Slug and **(E)** ZEB1 transcriptional repressors assessed in early- (Stages I-II) versus advanced-stage (Stages III-IV) serous ovarian tumors by quantitative real time PCR. Expression data correspond to the TCGA Ovarian Serous Cystadenocarcinoma database (***p<0.001, **p<0.01, *p<0.05, ns: no significant).

In addition to these studies, the mRNA expression levels of E-cadherin and the transcriptional repressors Twist, Snail, Slug and ZEB1, were evaluated in serous ovarian tumors of different FIGO stages from the information available at the TCGA data portal. Within a total of 564 tumor-tissue samples processed and analyzed, 534 entries were from primary tumors, 42 of which were from early stages and 492 from advanced stages. A large variability was observed for E-cadherin mRNA levels among samples of each group **(Fig 2A)**. In any case, E-cadherin transcript expression showed a trend toward decreased levels in advanced-stage tumors when compared to early-stage tumors (Stages I-II: 0.08991 ± 0.1081; Stages III-IV: 0.01496 ± 0.03958; p = 0.7885). This trend was in line with results of E-cadherin protein signal in the OC TMA **(Fig 1B and 1C)**. Moreover, a significant increase in the levels of Twist (p<0.001), Slug (p<0.01) and ZEB1 (p<0.05) repressors was observed in Stages III-IV tumors compared with those of Stages I-II **(Fig 2B, 2D and 2E)**. In contrast, Snail transcript levels were not significantly different (p = 0.2692, **Fig 2C**) among samples.

### Expression analyses of E-cadherin and EMT-related markers in OC cell lines. Its relationship with cell migration capacity

**Fig 3:**
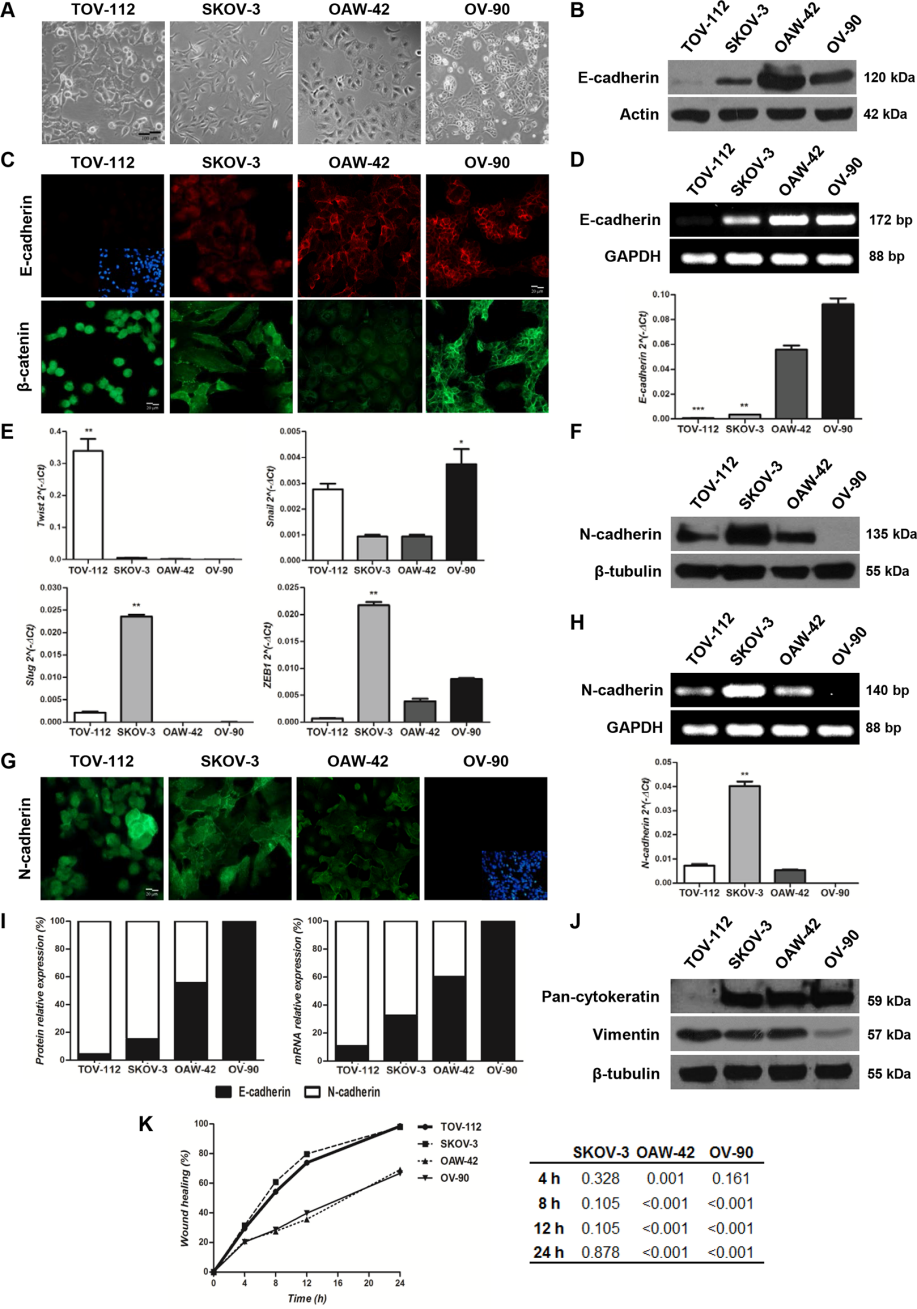
Expression analyses of E-cadherin and EMT-related markers in TOV-112, SKOV-3, OAW-42 and OV-90 OC cell lines. **Assessment of their migration capacity. (A)** Phase contrast images of cell lines grown in monolayers (100x magnification, scale bar 100 μm). **(B)** Western immunoblotting of the 120 kDa E-cadherin full lenght form. Actin was included for total protein loading control. **(C)** Immunofluorescence analyses of E-cadherin (top) and β-catenin (bottom) (400x magnification, scale bar 20 μm). Image of Hoechst 33342 nuclear staining was included for TOV-112 cells. **(D)** Standard (top) and quantitative real time (bottom) PCR analyses of E-cadherin mRNA expression. GAPDH was used as endogenous control (***p<0.001, **p<0.01). **(E)** mRNA expression levels of Twist, Snail, Slug and ZEB1 transcriptional repressors assessed by quantitative real time PCR (**p<0.01, *p<0.05). **(F)** Western immunoblotting analysis of N-cadherin. β-tubulin served as total protein loading control. **(G)** Fluorescent immunocytochemistry analysis of N-cadherin (400x magnification, scale bar 20 μm). Image of Hoechst 33342 nuclear staining was included for OV-90 cells. **(H)** Expression analysis of N-cadherin mRNA by both standard (top) and quantitative real time (bottom) PCR. GAPDH was used as endogenous control (**p<0.01). **(I)** Protein (left) and mRNA (right) relative expression (%) of E-cadherin (black) versus N-cadherin (white) in the 4 cell lines. **(J)** Western immunoblotting of pan-cytokeratin and vimentin. β-tubulin was included as total protein loading control. **(K)** Scratch-wound healing assay at 0, 4, 8, 12 and 24 hours. A graphical representation of wound healing values (%) as a function of time (h) is shown. A statistical analysis of OC cell lines migration rates at 4, 8, 12 and 24 hours was also included, considering the TOV-112 cell line as reference.

To further understand the implications of E-cadherin expression in OC progression, a set of studies were done with the OC cell lines TOV-112, SKOV-3, OAW-42 and OV-90. None of these cells were reported to have somatic mutation on *CDH1* [28]. A morphological analysis revealed striking differences among them: whereas TOV-112 and SKOV-3 cells showed spindle-shaped morphology with branched cytoplasm and low cellular contacts distinctive of fibroblast-like cells, OAW-42 and OV-90 cells depicted a more cuboidal shape with continuous cell-cell contacts and few intercellular spaces, a typical characteristic of epithelial cells **(Fig 3A)**.

When E-cadherin expression was analyzed by Western immunoblotting, TOV-112 cells depicted the lowest level of the 120 kDa full length (FL) form, while OAW-42 and OV-90 cells showed higher expression of E-cadherin than SKOV-3 cells **(Fig 3B)**. In agreement with these findings, immunocytochemical analysis of E-cadherin revealed no detectable levels of the adhesion protein in TOV-112 cells, mislocalization to the cellular cytoplasm in SKOV-3 cells, and plasma membrane localization in OAW-42 and OV-90 cells **(Fig 3C)**. Immunodetection of β-catenin showed plasma membrane localization of the adaptor protein in all cell lines expressing E-cadherin, as well as at the cytoplasm of TOV-112, SKOV-3 and OAW-42 cells **(Fig 3C)**. When analyzed at mRNA level, a lower E-cadherin expression was observed in TOV-112 compared to OV-90 and OAW-42 cells (p<0.001 and p<0.01, respectively), and in SKOV-3 compared to OV-90 cells (p<0.01) **(Fig 3D)**, in line with their E-cadherin protein levels **(Fig 3B)**.

Based on these results, the expression of the E-cadherin transcriptional repressors Twist, Snail, Slug and ZEB1 was evaluated by quantitative real time PCR **(Fig 3E)**. Whereas Twist showed the highest expression in TOV-112 (p<0.01), Slug and ZEB1 mRNA levels were highest in SKOV-3 cells (p<0.01). Furthermore, Snail depicted the highest expression levels in OV-90 cells (p<0.05) despite the high levels of the adhesion protein, suggesting a lack of E-cadherin regulation by this repressor in this cell line.

In addition to these evaluations, the expression of N-cadherin was studied in the above-mentioned OC cell lines. By Western immunoblotting, the 135 kDa FL N-cadherin form was detected in TOV-112, SKOV-3 and OAW-42 cell lines at variable levels, being the highest in SKOV-3 cells **(Fig 3F)**. Moreover, N-cadherin was immunolocalized at the cell membrane and cytoplasm of TOV-112, SKOV-3 and OAW-42 cells, while OV-90 showed no N-cadherin signal **(Fig 3G)**. The same trend was observed for the N-cadherin transcript, showing highest levels in SKOV-3 cells (p<0.01) **(Fig 3H)**. When the relative expression of E- to N-cadherin was analyzed at protein and mRNA levels, these molecules showed a distinct proportion in the 4 cell lines **(Fig 3I)**. To further characterize the molecular phenotype, the expression of cytokeratins (epithelial markers) and vimentin (mesenchymal marker) was also evaluated by Western immunoblotting in the OC cell lines **(Fig 3J)**. As a result, TOV-112 cells expressed high levels of vimentin and OV-90 depicted high levels of cytokeratins, while SKOV-3 and OAW-42 cells showed high expression levels of both markers.

The expression levels of E- and N-cadherin, together with cytokeratins and vimentin (EMT profile), led us to classify the OC cell lines as mesenchymal (M; TOV-112), intermediate (I; SKOV-3 and OAW-42) and epithelial (E; OV-90). Furthermore, SKOV-3 and OAW-42 cells were sub-classified as intermediate mesenchymal (IM; SKOV-3) and intermediate epithelial (IE; OAW-42), based on the E- and N-cadherin levels. These phenotypes were previously described by Wang and collaborators [29], although using different criteria.

Since the expression of mesenchymal markers has been associated with a more motile and invasive cell behavior [12], the migration capacity of cell lines was evaluated by the wound-healing assay. Consistent with the previous results, a statistical analysis revealed higher migration rates for TOV-112 and SKOV-3 than for OAW-42 at 4 hours (p = 0.001), and for OAW-42 and OV-90 cells at 8, 12 and 24 hours (p<0.001) **(Fig 3K)**. Moreover, M (TOV-112) and IM (SKOV-3) cells were able to close the wound within 24 hours **(Fig 3K)**, while IE (OAW-42) and E (OV-90) cells needed additional 24 hours to heal the lesion **(S3 Fig)**.

### Aggregation and survival of OC cell lines grown under anchorage-independent conditions

**Fig 4:**
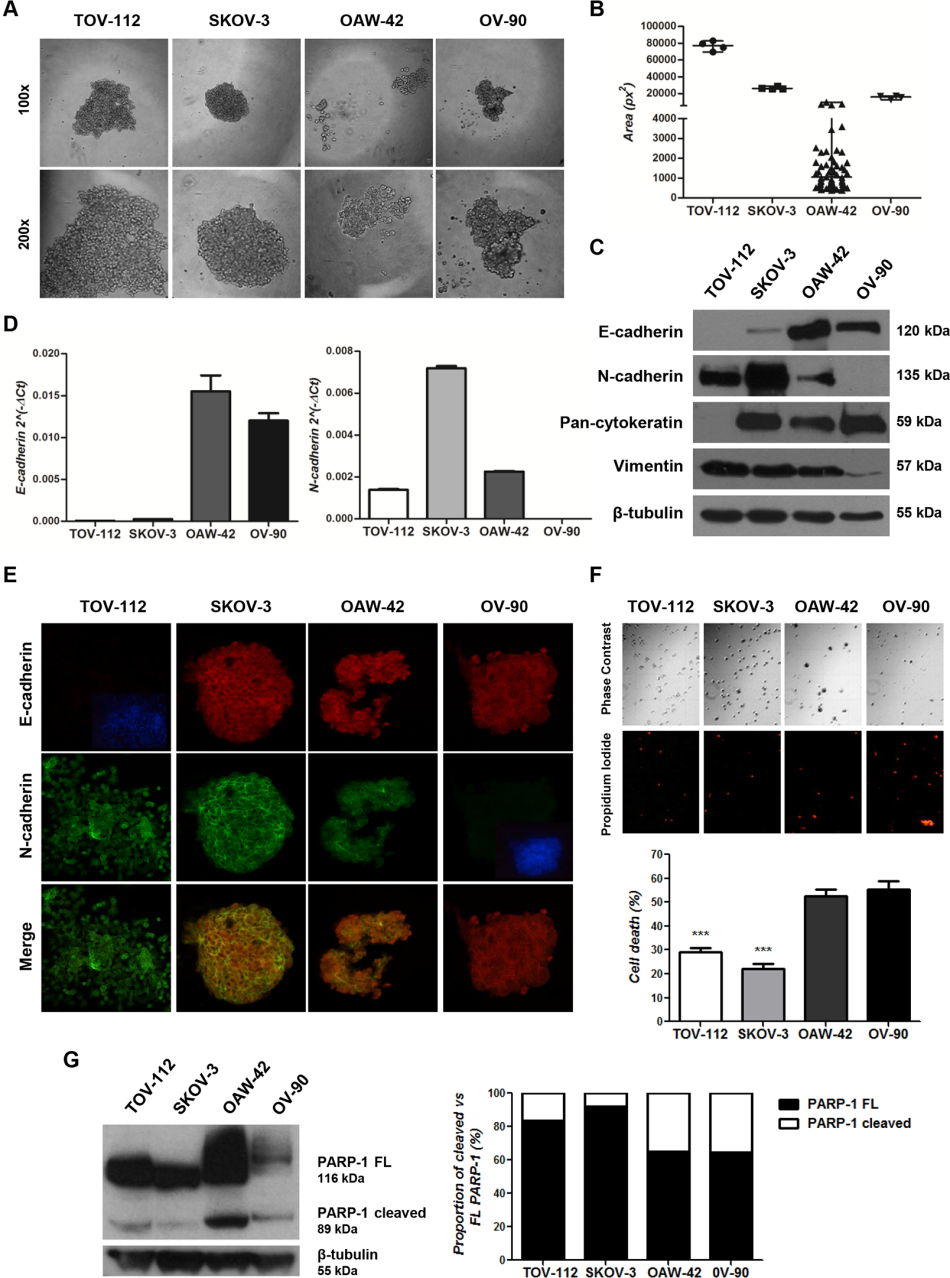
Expression analyses of E-cadherin and EMT-related markers in OC cell lines grown under anchorage-independent conditions. **Assessment of their aggregation and survival capacities. (A)** Phase contrast images of 48 hour-aggregates (100x and 200x magnifications). **(B)** Plot of the area (px^2^: pixeles^2^) and number (black spots) of 48 hour-aggregates in 4 drops of each cell line. **(C)** Western immunoblotting analyses of E-cadherin, N-cadherin, pan-cytokeratin and vimentin in 48 hour-aggregates. β-tubulin served as total protein loading control. **(D)** Quantitative real time PCR analyses of E-cadherin and N-cadherin mRNA expression levels in 48 hour-aggregates. **(E)** Fluorescent immunocytochemistry analysis of E-cadherin and N-cadherin in 48 hour-aggregates (400x magnification). A merge image of both cadherins is also included. **(F)** Cell death assessed by means of PI staining in 48 hour-aggregates. Images were taken using an inverted microscope with phase contrast and red fluorescence after PI staining of disaggregated cells (100x magnification) (top). Cell death (%) was plotted (bottom) (***p<0.001). **(G)** Western immunoblotting analysis of PARP-1 on 48 hour-aggregates (left). Relative expression (%) of cleaved versus FL PARP-1 form (right).

OC dissemination involves primary tumor cell exfoliation, release into the peritoneal cavity and survival. These cells can also form multicellular aggregates in suspension and then give rise to a metastatic implant [4]. Based on this background information, TOV-112, SKOV-3, OAW-42 and OV-90 cells were grown under anchorage-independent conditions by the hanging drop method to mimic this dissemination process. As shown in **S4 Fig**, TOV-112, SKOV-3 and OV-90 cell lines aggregated in large multicellular structures at 24 hours, which converged in a single one after 48 hours. In contrast, OAW-42 cells did not form single large structures at any time evaluated; instead, small-scattered aggregates were found at 24 hours **(S4 Fig)** and “grape-like” aggregates of irregular shape were observed after 48 hours. Representative images of the 48 hour-multicellular structures for all cell lines are shown in **Fig 4A**. The area and the number of aggregates were analyzed in four 48 hour-drops of each cell line **(Fig 4B)**.

Despite the ability of TOV-112, SKOV-3 and OV-90 cells to form a single aggregate after 48 hour culture, cell compaction appeared slightly different between TOV-112 and OV-90 compared with SKOV-3 cells. While smooth contoured structures with tightly packed cells and lack of intercellular spaces was observed in IM (SKOV-3) cells, less compacted aggregates were found in the other cell lines. Furthermore, the E (OV-90) cell drops showed a higher number of single cells, suggesting a lower capacity to maintain the aggregate structure for this cell line **(Fig 4A)**.

To better understand the molecular basis of these OC cell-aggregates, the expression of E- and N-cadherin (protein and mRNA levels), cytokeratins and vimentin (protein level), were evaluated **(Fig 4C–4E)**. Protein expression assessed by Western immunoblotting and immunofluorescence analyses (**Fig 4C and 4E**, respectively) showed the same phenotypes in the cell-aggregates as in cells grown in monolayers. Regarding mRNA studies, E- and N-cadherin expression profiles were comparable among the different cell lines grown in both conditions (**Fig 4D** versus **Fig 3D and 3H**).

To characterize the aggregates behavior, cell death was evaluated by means of 2 experimental approaches. When the percentage (%) of cell death in 48 hour-aggregates was determined by the PI assay, aggregates of TOV-112 and SKOV-3 cells showed lower percentage of dead cells (28.9% and 22.1%, respectively) than OAW-42 and OV-90 cell lines (52.6% and 55.2%, respectively) (p<0.001) **(Fig 4F)**. On the other hand, among cell aggregates depicting the highest survival rates, those derived from IM cells showed a lower (p<0.05) cell death than those with an M phenotype. Representative images of phase contrast and PI-stained cells from the disaggregated structures are also shown **(Fig 4F)**.

As a second approach, the expression of PARP-1 protein was evaluated **(Fig 4G)**. Since PARP-1 is cleaved by caspases in cells undergoing apoptosis, presence of the 116 kDa FL and the 89 kDa cleaved PARP-1 forms was analyzed. When the proportion of cleaved to FL PARP-1 was plotted, TOV-112 and SKOV-3 showed a lower percentage of cleaved PARP-1 (16.6% and 7.9%, respectively) than OAW-42 and OV-90 aggregates (35% and 35.5%, respectively). Interestingly, between aggregates with a mesenchymal-like phenotype, those from IM (SKOV-3) cells exhibited the lowest rate of PARP-1 cleavage, in line with results from the PI assay.

### Adhesion, disaggregation and invasion capacity of OC cell aggregates

Since OC dissemination involves adhesion, disaggregation and invasion of cell aggregates at local pelvic and abdominal organs [30], the adhesion capacity of 48 hour-aggregates from OC cell lines to fibronectin and collagen I was initially evaluated.

**Fig 5:**
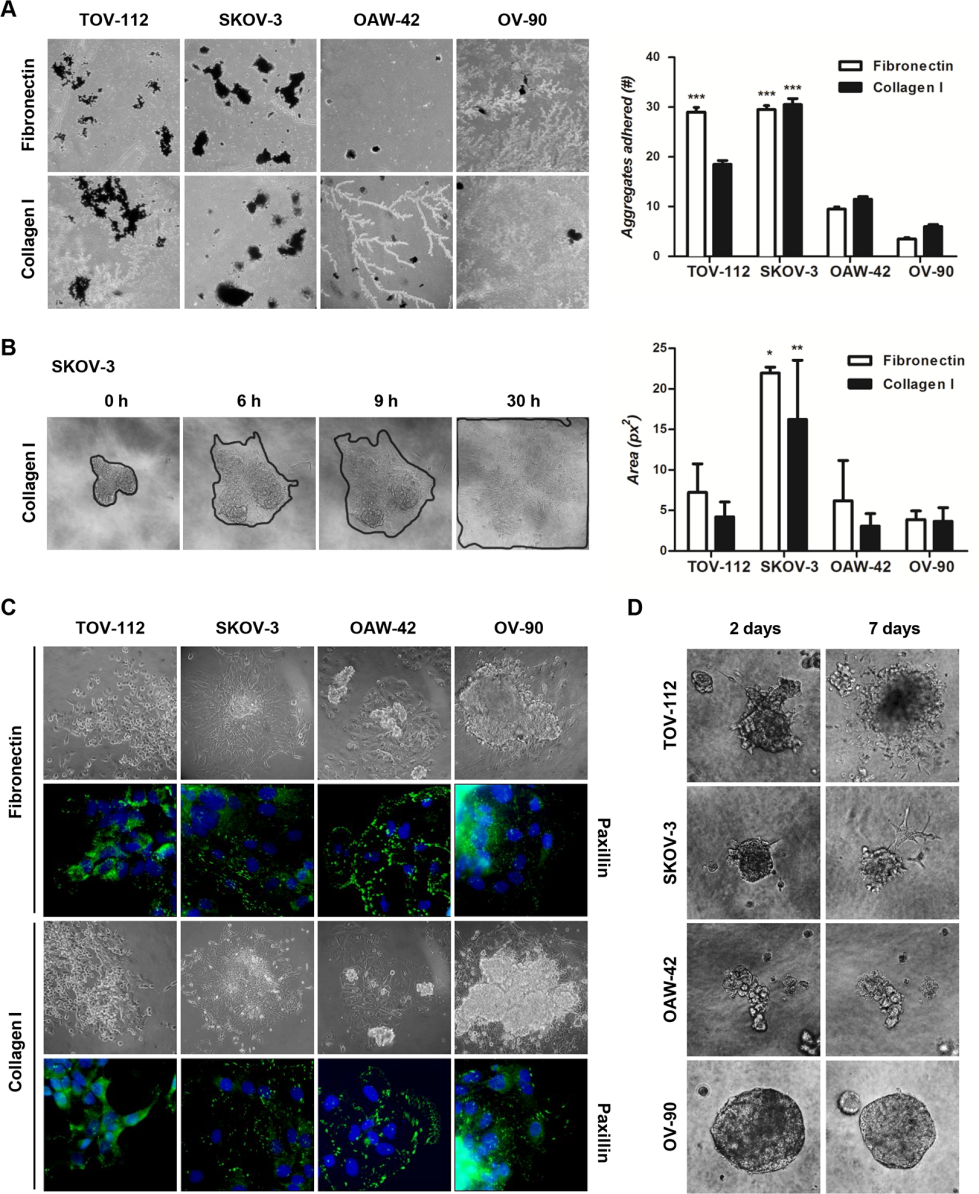
Assessment of adhesion, disaggregation and invasion capacity of OC cell lines grown under anchorage-independent conditions. **(A)** Representative phase contrast images of 48 hour-aggregates (black spots) placed onto fibronectin and collagen I matrices (40x magnification) (left). The number (#) of aggregates adhered to each matrix (Fibronectin: white, Collagen I: black) after 2 hour-incubation was plotted (right) (***p<0.001). **(B)** Representative phase contrast images of the area of an SKOV-3 aggregate placed onto collagen I over time (0, 6, 9, 30 hours) are shown (left) (100x magnification). Estimated area (px^2^: pixeles^2^) of 48 hour-aggregates placed onto fibronectin (white) and collagen I (black) for 30 hours (right) (**p<0.01, *p<0.05). **(C)** Phase contrast images and immunofluorescence analysis of paxillin in 48 hour-aggregates placed onto fibronectin and collagen I for 24 hours (200x and 400x magnification, respectively). **(D)** Phase contrast images of 48 hour-aggregates 2 and 7 days after placing them into Matrigel^TM^ (200x magnification).

TOV-112 and SKOV-3 showed a higher number of aggregates adhered to fibronectin compared to OAW-42 and OV-90 (p<0.001). Among the mesenchymal-like aggregates, those derived from IM (SKOV-3) cells showed the highest adhesion capacity to collagen I (p<0.001) **(Fig 5A)**.

To assess disaggregation after adhesion to both ECM, the area of the aggregate structures was recorded over time up to 30 hours. Representative images of disaggregation of a SKOV-3 aggregate onto collagen I are shown in **Fig 5B**. As a result, IM cell-aggregates displayed the largest area onto collagen I at 24 hours (p<0.01; **S5 Fig**) and onto both ECM at 30 hours (Fibronectin: p<0.05; Collagen I: p<0.01) **(Fig 5B and S5 Fig)**. In addition, after 24 hours of interaction between aggregates and both ECM, immunolocalization analysis of paxillin revealed the presence of this protein in aggregates from all OC cell lines **(Fig 5C)**. Paxillin is a focal adhesion protein involved in the structural link between ECM and the actin cytoskeleton [31]. Thus, despite being adhered, TOV-112, OAW-42 and OV-90 aggregates do not have the ability to disseminate onto the ECM.

To further evaluate the invasive behavior of cell aggregates, the 3D-Matrigel^TM^ assay was performed. Representative images of 2 and 7 day-aggregates from OC cell lines into Matrigel^TM^ are shown in **Fig 5D**. No signs of invasion were observed in OAW-42 and OV-90 aggregates at any time analyzed. In contrast, while TOV-112 aggregates showed individual cells randomly spreading out of them, SKOV-3 structures displayed typical invasive branches [32].

### Evaluation of E-cadherin and EMT-related markers in human serous ovarian tumor- and ascites-primary cultures

In order to translate these *in vitro* findings to the bedside, E-cadherin, N-cadherin, cytokeratin 19 and vimentin mRNA expression levels were evaluated in 6 tumor- and 20 ascites-primary cultures derived from patients diagnosed with advanced-stage high-grade serous OC **(Tables 2 and 3)**.

**Table 2:** E-cadherin, N-cadherin, cytokeratin 19 and vimentin mRNA expression levels in human ovarian tumor-primary cultures.

*Sample*	*E-cadherin 2^(-ΔCt)*	*N-cadherin 2^(-ΔCt)*	*Cytokeratin 19 2^(-ΔCt)*	*Vimentin 2^(-ΔCt)*
Primary Tumor	0.001987	0.206613	0.212421	0.411796
Primary Tumor	0.002850	0.246558	0.547147	1.172835
Primary Tumor	0.003594	0.357249	0.012736	0.020617
Primary Tumor	0.004143	0.326465	0.017337	0.049037
Primary Tumor	0.011203	0.343885	0.214641	0.230047
Primary Tumor	0.013139	0.161544	0.006778	0.017098

**Table 3:** E-cadherin, N-cadherin, cytokeratin 19 and vimentin mRNA expression levels in human OC ascites-primary cultures.

*Sample*	*Sample Name*	*E-cadherin 2^(-ΔCt)*	*N-cadherin 2^(-ΔCt)*	*Cytokeratin 19 2^(-ΔCt)*	*Vimentin 2^(-ΔCt)*
Ascites	A1	0.000114	0.166662	0.022000	0.280292
Ascites	A2	0.000369	0.171943	0.202000	0.524858
Ascites	A3	0.000750	0.288172	0.091500	0.380245
Ascites	A4	0.000883	0.129408	0.044800	0.539614
Ascites	A5	0.001084	0.291183	0.091800	0.441351
Ascites	A6	0.003023	0.210224	0.265000	0.523042
Ascites	A7	0.003460	0.250868	0.153000	0.580352
Ascites	A8	0.005411	0.208050	0.049037	0.240649
Ascites	A9	0.005921	0.294227	0.526681	0.719467
Ascites	A10	0.006592	0.318640	0.203768	0.458502
Ascites	A11	0.006992	0.127627	0.042986	0.299370
Ascites	A12	0.007652	0.141610	0.031034	0.188809
Ascites	A13	0.008549	0.175556	0.423373	0.246558
Ascites	A14	0.008881	0.235696	0.102238	0.558644
Ascites	A15	0.011883	0.085971	0.022021	0.142102
Ascites	A16	0.017337	0.168404	0.003545	0.326465
Ascites	A17	0.025916	0.218393	0.089622	0.381565
Ascites	A18	0.026645	0.158769	0.052922	0.222982
Ascites	A19	0.029873	0.180491	0.038607	0.199575
Ascites	A20	0.032690	0.295248	0.018389	0.170755

**Fig 6:**
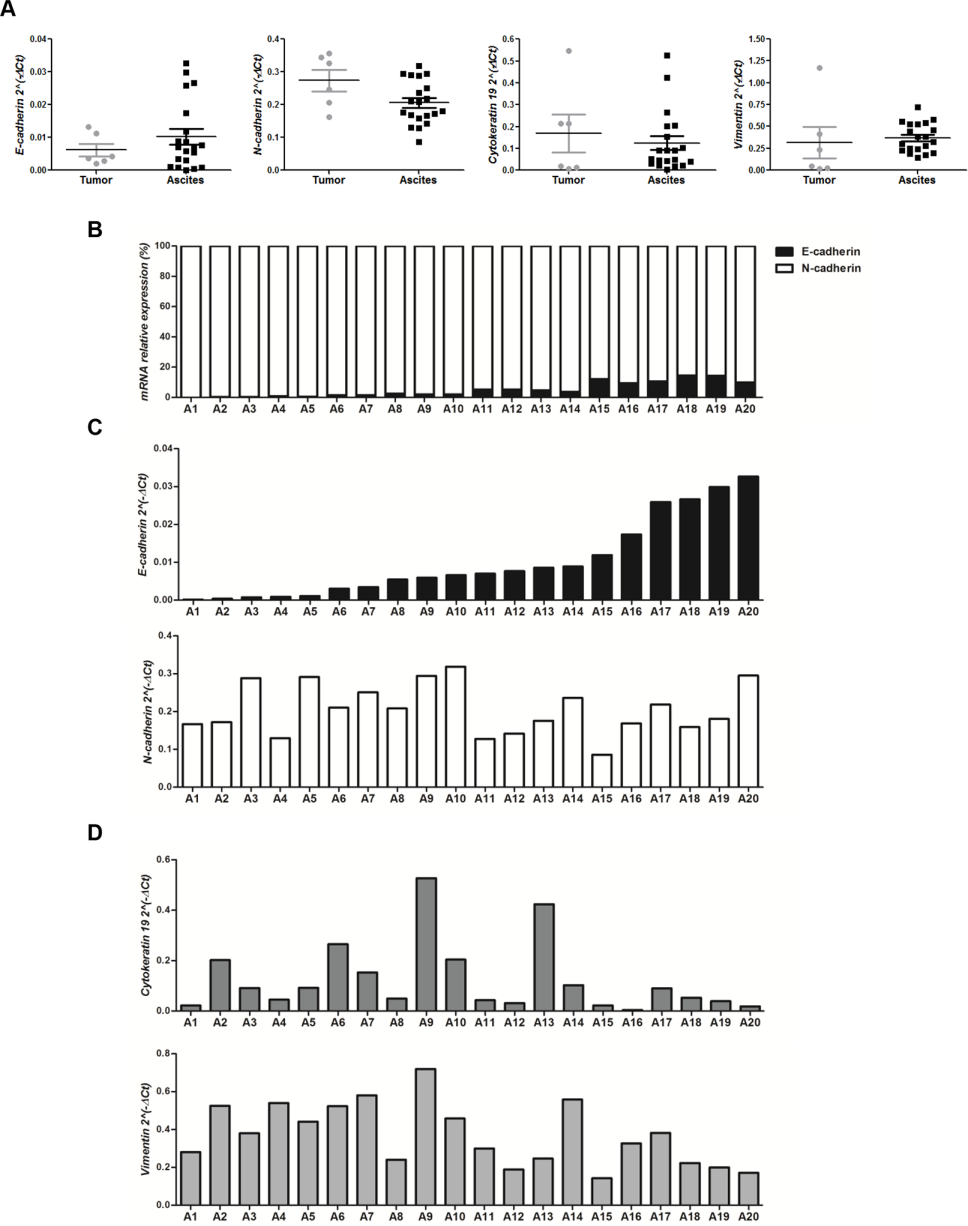
Expression analyses of E-cadherin and EMT-related markers in human advanced-stage high-grade serous OC tumor- and ascites-primary cultures. **(A)** mRNA expression levels of E-cadherin, N-cadherin, cytokeratin 19 and vimentin, assessed in 6 OC tumor- versus 20 ascites-primary cultures by quantitative real time PCR (ns: not significant). **(B)** mRNA relative expression (%) of E-cadherin (black) versus N-cadherin (white) in 20 ascites-primary cultures. **(C and D)** Quantitative real time PCR analyses of **(C)** E-cadherin (top) and N-cadherin (bottom), and **(D)** cytokeratin 19 (top) and vimentin (bottom), mRNA expression levels in 20 ascites-primary cultures.

The 4 mRNAs were detected in all samples analyzed. Regarding E-cadherin expression, a trend towards a higher mean value in ascites compared to tumors (0.010201 versus 0.006153; 1.66x) was found. In contrast, differences not higher than 0.25x were observed in the mean value of N-cadherin (0.205857 versus 0.273719; 0.75x), cytokeratin 19 (0.123716 versus 0.168510; 0.73x) and vimentin (0.371260 versus 0.316905; 1.17x) mRNA levels in ascites versus tumors **(Fig 6A, Tables 2 and 3)**.

Among ascites, when the relative abundance of both cadherins was calculated for each sample, a higher proportion of N- to E-cadherin mRNA was observed in all cases **(Fig 6B)**. E-cadherin mRNA levels showed a large dispersion among samples, with values that ranged from 0.00011 to 0.03269 (297x increase). In contrast, N-cadherin levels varied up to a 3.71x increase (from 0.08597 to 0.31864) **(Fig 6C and Table 3)**. On the other hand, whereas cytokeratin 19 mRNA values varied more than one hundred times (from 0.00355 to 0.52668; a 148.36x increase), vimentin values increased up to 5.06 times (from 0.14210 to 0.71947) **(Fig 6D and Table 3)**.

Since in the ascitic samples a large dispersion of mRNA values was only observed for the epithelial markers E-cadherin and cytokeratin 19, the relationship between these values and those of 2 clinicopathological parameters indicative of OC aggressiveness (CA125 and PFI) was evaluated by performing a correlation analysis. As a result, only E-cadherin mRNA levels showed a significant correlation with both CA125 and PFI values (E-cadherin: CA125: r = 0.5113, p = 0.0212 and PFI: r = -0.4883, p = 0.0289; cytokeratin 19: CA125: r = -0.09925, p = 0.6772 and PFI: r = 0.2011, p = 0.3953). Moreover, considering the median value (median = 0.00679) of E-cadherin mRNA levels, samples were divided in 2 groups: low (A1-A10) and high (A11-A20) E-cadherin expression. Samples with lower E-cadherin levels than the median value showed CA125 levels less than 500 U/mL in 7/10 cases and PFI greater than 6 months in 7/10 cases. Contrasting, those with E-cadherin mRNA expression higher than the median value were associated to CA125 levels greater than 500 U/mL in 7/10 cases and a PFI less than 6 months in 7/10 cases **(Table 4)**.

**Table 4:** E-cadherin mRNA expression, CA125 levels and PFI in OC ascites.

*Sample Name*	*E-cadherin 2^(-ΔCt)*	*CA125 (U/mL)*	*PFI (months)*
A1	0.000114	72	9
A2	0.000369	559	14
A3	0.000750	112	7
A4	0.000883	42	10
A5	0.001084	223	14
A6	0.003023	82	26
A7	0.003460	217	18
A8	0.005411	1217	4
A9	0.005921	4857	2
A10	0.006592	417	6
A11	0.006992	123	4
A12	0.007652	6049	4
A13	0.008549	26	< 3
A14	0.008881	7416	10
A15	0.011883	363	3
A16	0.017337	777	< 3
A17	0.025916	908	9
A18	0.026645	1560	during treatment
A19	0.029873	760	1
A20	0.032690	580	14

## Discussion

OC is frequently referred to as a “silent killer”, because most cases are diagnosed at advanced stages when the tumor has already metastasized into the peritoneal cavity. Several studies have described the deregulation of E-cadherin mediated cell-cell adhesion and EMT-related molecules in OC progression and dissemination [19, 33, 34]. However, no conclusive findings have been reported on the impact of their expression in tumor agressiveness and patient prognosis. The present study was focused on the characterization of E-cadherin expression levels in human ovarian tumors to relate them with the main clinicopathological parameters. In addition, the expression levels of 4 EMT-markers (E-cadherin, N-cadherin, cytokeratin 19 and vimentin) were evaluated in human OC ascites- and tumor-primary cultures. These markers were selected based on the molecular and functional characterization of an *in vitro* model carried out using cell lines grown under anchorage-independent conditions, an experimental approach that resembles OC dissemination into the peritoneal cavity. To evaluate their impact in tumor aggressiveness, the relationship between mRNA expression levels of the selected EMT-markers and patient prognosis, measured by means of CA125 levels and PFI, was determined.

Firstly, E-cadherin protein levels and subcellular localization were evaluated by immunohistochemistry in 76 human ovarian tumors arranged in a TMA. A relationship between total and membranous E-cadherin low levels and OC poor prognosis was observed, in line with previous studies [35, 36]. In fact, both E-cadherin measurements were found to be good markers to differentiate between advanced- and early-stage ovarian tumors. Moreover, they differentiated serous tumors, the most frequent and aggressive histological type, from other histologies, in agreement with a recent study using an OC TMA that evaluated only the membranous E-cadherin signal [37]. Unlike total and membranous E-cadherin expression, no relationship was found between cytoplasmic localization and any clinicopathological parameter analyzed. Furthermore, nuclear E-cadherin expression was associated only with tumor grade, finding a higher proportion of low-grade tumors depicting this signal than high-grade tumors. In this regard, some authors have reported the association of an aberrant nuclear E-cadherin signal with a negative regulation of the Wnt/β-catenin pathway and a better prognosis in other carcinomas [38]. Finally, our study is the first one reporting results on sensitivity and specificity data analyses, which revealed the importance on the assessment of both total and membranous E-cadherin expression to distinguish advanced versus early ovarian tumors, as well as serous tumors from other histological types.

The decreased E-cadherin protein expression observed in advanced- versus early-stage serous ovarian tumors was also revealed at transcript level in a TCGA database of over 500 cases. These findings could be associated, at least in part, with a significant increase in Twist, Slug and ZEB1 mRNA levels with tumor progression, in accordance with reports that describe the expression of these E-cadherin transcriptional repressors in OC [39–41]. Taking into account the low rate of somatic mutations found in over 700 ovarian serous tumors listed in COSMIC, transcriptional regulation of E-cadherin levels is a relevant mechanism in OC progression.

The low E-cadherin expression observed in advanced-stage tumors would be in favor of OC dissemination by direct extension of tumor cells into the peritoneal cavity. Nevertheless, the relationship between E-cadherin levels and malignancy of detached OC cells is yet controversial: both high and low E-cadherin expression levels have been described [20, 29, 42–44]. To address this issue, an *in vitro* model was established using the TOV-112, SKOV-3, OAW-42 and OV-90 OC cell lines. The molecular characterization studies performed in these cells grown in monolayers showed differences in E-cadherin expression levels among them, which were associated with the expression of transcriptional repressors. In particular, Twist, Slug and ZEB1 were the highest ones expressed in cell lines depicting the lowest E-cadherin levels (TOV-112 and SKOV-3). These findings agree with those from the TCGA database analysis, in favor of using these cell lines to study regulation of E-cadherin expression in OC progression.

Besides E-cadherin analysis, protein expression of N-cadherin, cytokeratins and vimentin led us to classify the 4 OC cell lines in different EMT profiles: mesenchymal (M; TOV-112), intermediate (I; SKOV-3 and OAW-42), and epithelial (E; OV-90). Moreover, based on the relative proportion of E- to N-cadherin expression levels, cells were subclassified into intermediate mesenchymal (IM; SKOV-3) and intermediate epithelial (IE; OAW-42) subtypes. Although terms selected to define these 4 phenotypes were previously used by Huang and colleagues [29], in that report the M and E phenotypes were based only on the absence or presence of E-cadherin, respectively, and the IM and IE were defined by the expression of vimentin and cytokeratins.

When grown under anchorage-independent conditions, the 4 OC cell lines were able to form cellular aggregates that showed the same EMT profiles found in cells grown in monolayers, but differences in their behavior were observed. The assessment of cell death, cell-matrix adhesion and cell invasiveness showed that aggregates with an M-like phenotype (M: TOV-112; IM: SKOV-3) had a higher survival rate as well as an increased ability to attach to fibronectin and collagen I ECM and to invade through Matrigel™ than those with an E-like phenotype (E: OV-90; IE: OAW-42). In line with our results, an enrichment of mesenchymal genes was reported in OC cell lines and human samples depicting an invasive phenotype [45]. Interestingly, between M-like aggregates, those derived from SKOV-3 cells (IM phenotype) depicted a higher ability to survive in suspension, to adhere and disaggregate onto both ECM than TOV-112 aggregates (M phenotype). Altogether, the use of OC cell lines grown in suspension revealed an association between the highest cell aggressive behavior and the IM molecular phenotype, characterized by a high proportion of N- to E-cadherin expression in addition to the co-expression of cytokeratins and vimentin.

Studies assessing the EMT profile in 20 ascites- and 6 tumor-primary cultures derived from patients diagnosed with advanced-stage high-grade serous OC, showed the expression of E- and N-cadherin, cytokeratin 19 and vimentin mRNA in all samples. The functional consequences of the co-expression of these EMT markers in OC cells would be in favor of the concept of cell plasticity, which has been related to cell aggressiveness [29, 46]. Interestingly, among the 4 markers, E-cadherin showed the highest increased mRNA expression (around 70%) in ascites- compared to tumor-primary cultures, suggesting a potential role of this molecule in the OC cell dissemination process.

A further analysis focused on the relative proportion of N- to E-cadherin and the presence of both vimentin to cytokeratin 19 mRNA in ascites, revealed an IM phenotype in all samples evaluated. Furthermore, the quantification analysis of the 4 EMT markers showed a low dispersion of N-cadherin and vimentin mRNA expression, contrasting with the high dispersion found among mRNA levels recorded for the epithelial markers. However, E-cadherin mRNA levels were the only ones that significantly correlated with CA125 levels and PFI, both measurements used worldwide for OC patient prognosis.

**Fig 7:**
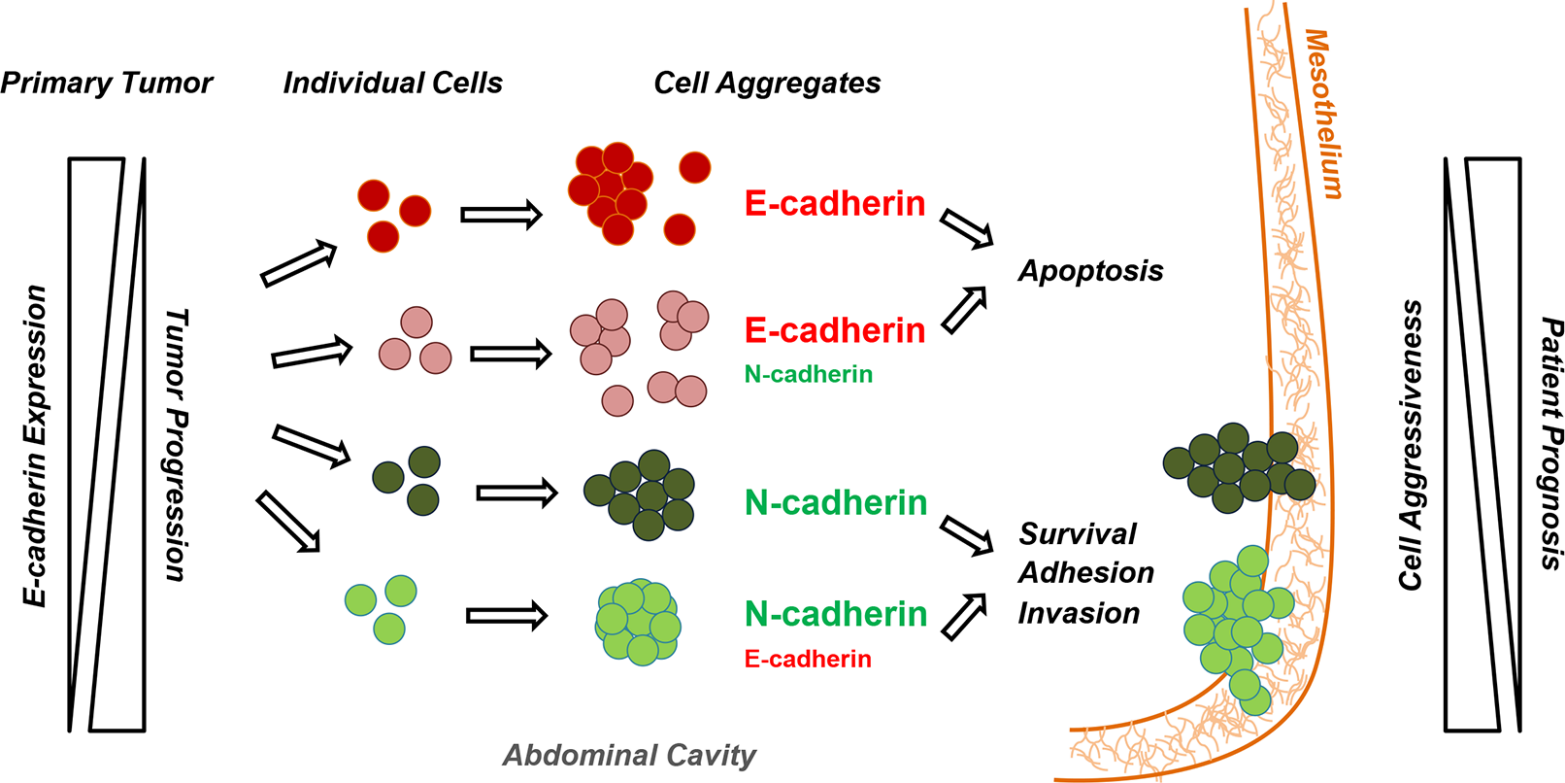
Schematic representation of the OC dissemination process. Along epithelial ovarian tumor progression E-cadherin expression levels decrease with tumor FIGO stages, contributing to the shedding of OC cells into the abdominal cavity. Individual cells could then aggregate in suspension and form multicellular structures with different expression levels of E- and N-cadherin, as well as of cytokeratins and vimentin. According to the expression of these EMT-related markers, cell aggregates could be classified as mesenchymal (M; expression of N-cadherin and vimentin, and absence of E-cadherin and cytokeratins detectable levels), intermediate mesenchymal (IM; expression of N-cadherin, E-cadherin, cytokeratins and vimentin, with a high N- to E-cadherin proportion), intermediate epithelial (IE; expression of N-cadherin, E-cadherin, cytokeratins and vimentin, with a high E- to N-cadherin proportion) and epithelial (E; expression of E-cadherin and cytokeratins, and absence of N-cadherin and vimentin detectable levels).Those aggregates with an mesenchymal-like phenotype (M and IM) will be able to survive under anchorage-independent conditions and to adhere and invade the mesothelium lining, leading to metastasis and a worse patient prognosis. However, aggregates with an epithelial-like phenotype (E and IE) will be more prompted to undergo apoptosis.

Based on our findings, a model describing changes in expression levels of E-cadherin in the primary tumor and in disseminating cells is shown in **Fig 7**. A decrease in E-cadherin expression is associated with epithelial ovarian tumor progression, contributing to the shedding of OC cells into the abdominal cavity. Individual cells then aggregate in suspension and form multicellular structures with different expression levels of E-cadherin, as well as N-cadherin, cytokeratins and vimentin. Cell aggregates with an epithelial-like phenotype (E and IE) are more prompted to undergo apoptosis, whereas those classified as mesenchymal-like (M and IM) are able to survive under anchorage-independent conditions and to adhere and invade the mesothelium lining, leading to metastasis and a worse patient prognosis.

In summary, results from the present study have demonstrated that E-cadherin is a determinant molecule associated with OC progression, dissemination and aggressiveness. For the first time, we have shown that total and membranous E-cadherin expression levels is a specific and sensitive marker to differentiate advanced- from early-stage tumors and serous tumors from other histological types. Moreover, E-cadherin mRNA expression levels in ovarian cancer ascites depicting an IM phenotype is a predictive marker of OC patient prognosis.

## Supporting information

S1 Fig Comparison of E-cadherin mRNA expression levels between single and pooled samples of TOV-112, SKOV-3, OAW-42 and OV-90 OC cell lines grown in monolayers. E-cadherin mRNA expression analysis of **(A)** single and **(B)** pooled cell lines samples by quantitative real time PCR.
(TIF)

S2 Fig Expression analysis of E-cadherin protein in a human OC TMA and its association with clinicopathological parameters. **(A)** Representative images of E-cadherin staining for early-stage (Stage I: serous, mucinous, endometrioid, clear cell; Stage II: undifferentiated) and advanced-stage (Stage III in all cases) tumors of different histological types (100x and 1000x magnifications). **(B-E)** Protein expression analysis of **(B)** total, **(C)** membranous, **(D)** cytoplasmic and **(E)** nuclear E-cadherin in 76 ovarian tumors arranged in a TMA, and the relationship with tumor stage, grade and histology.
(TIF)

S3 Fig Wound healing assay. Representative phase contrast images (100x magnification) of TOV-112, SKOV-3, OAW-42 and OV-90 cell lines, 0, 4, 8, 12 and 24 hours (h) after making the heal. For OAW-42 and OV-90 images are also shown 48 h after making the heal.
(TIF)

S4 Fig Morphological analysis of TOV-112, SKOV-3, OAW-42 and OV-90 aggregates of 24 hours. Representative phase contrast images (100x and 200x magnifications) of TOV-112, SKOV-3, OAW-42 and OV-90 24 hour-aggregates generated by the hanging drop method.
(TIF)

S5 Fig Disaggregation assay. **(A)** Representative phase contrast images (100x and 200x magnifications) of TOV-112, SKOV-3, OAW-42 and OV-90 aggregates, disaggregating onto fibronectin and collagen I matrices after 30 hours. **(B)** Graphical representation of the area (px^2^: pixeles^2^) of TOV-112, SKOV-3, OAW-42 and OV-90 aggregates disaggregating onto fibronectin (left) and collagen I (right) as a function of time (h).
(TIF)
