# Supplementary figures and images for: E-cadherin: A determinant molecule associated with ovarian cancer progression, dissemination and aggressiveness
Source: PLoS One. 2017 Sep 21;12(9):e0184439. doi: 10.1371/journal.pone.0184439 (PMC5608212; doi:10.1371/journal.pone.0184439)

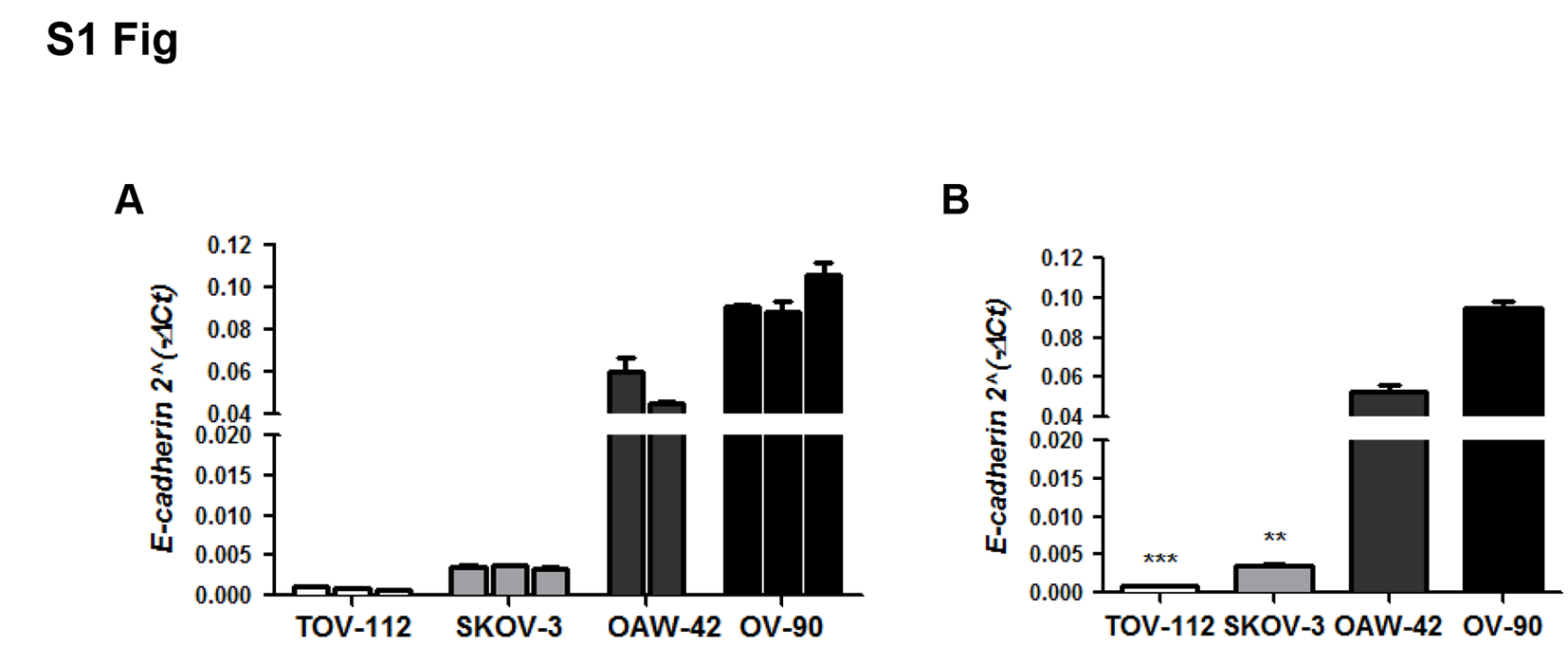

Supplement: S1 Fig — E-cadherin mRNA expression analysis of (A) single and (B) pooled cell lines samples by quantitative real time PCR. (TIF) [file pone.0184439.s001.tif]

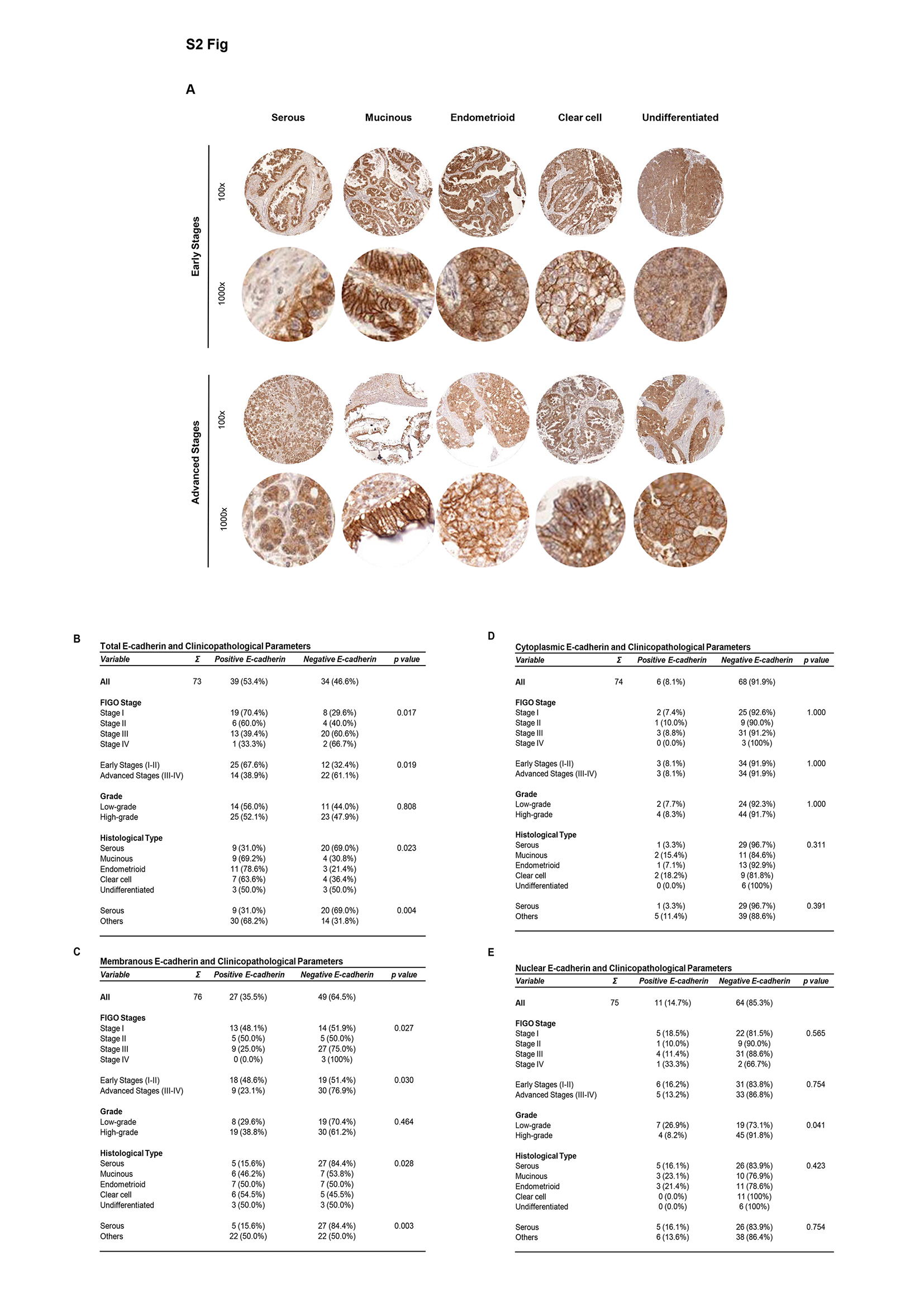

Supplement: S2 Fig — (A) Representative images of E-cadherin staining for early-stage (Stage I: serous, mucinous, endometrioid, clear cell; Stage II: undifferentiated) and advanced-stage (Stage III in all cases) tumors of different histological types (100x and 1000x magnifications). (B-E) Protein expression analysis of (B) total, (C) membranous, (D) cytoplasmic and (E) nuclear E-cadherin in 76 ovarian tumors arranged in a TMA, and the relationship with tumor stage, grade and histology. (TIF) [file pone.0184439.s002.tif]

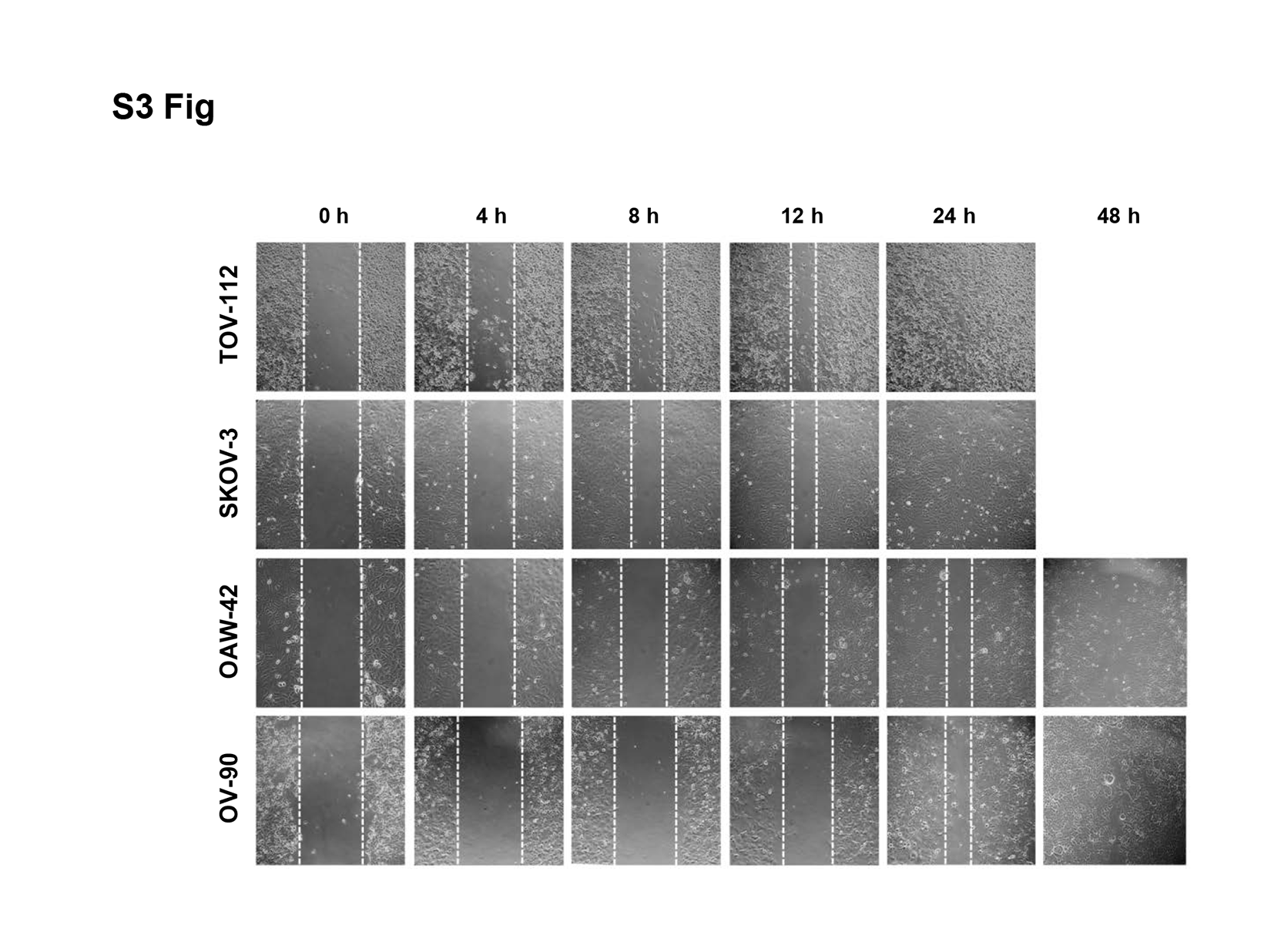

Supplement: S3 Fig — Representative phase contrast images (100x magnification) of TOV-112, SKOV-3, OAW-42 and OV-90 cell lines, 0, 4, 8, 12 and 24 hours (h) after making the heal. For OAW-42 and OV-90 images are also shown 48 h after making the heal. (TIF) [file pone.0184439.s003.tif]

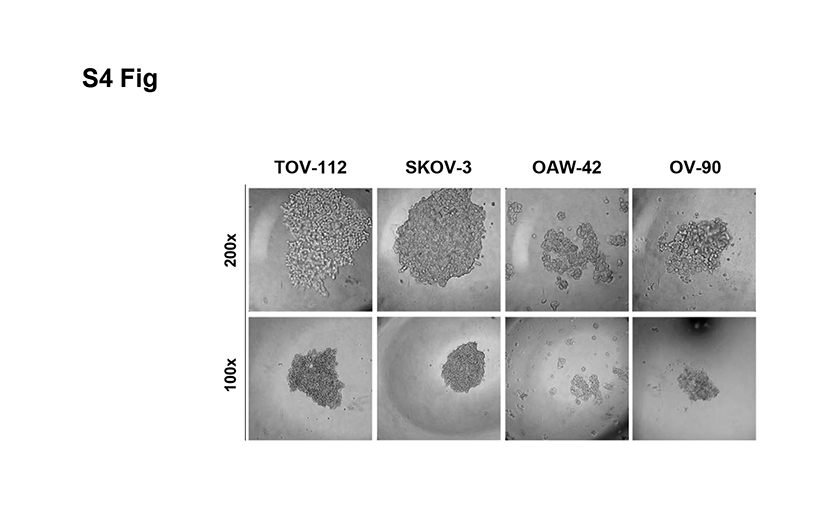

Supplement: S4 Fig — Representative phase contrast images (100x and 200x magnifications) of TOV-112, SKOV-3, OAW-42 and OV-90 24 hour-aggregates generated by the hanging drop method. (TIF) [file pone.0184439.s004.tif]

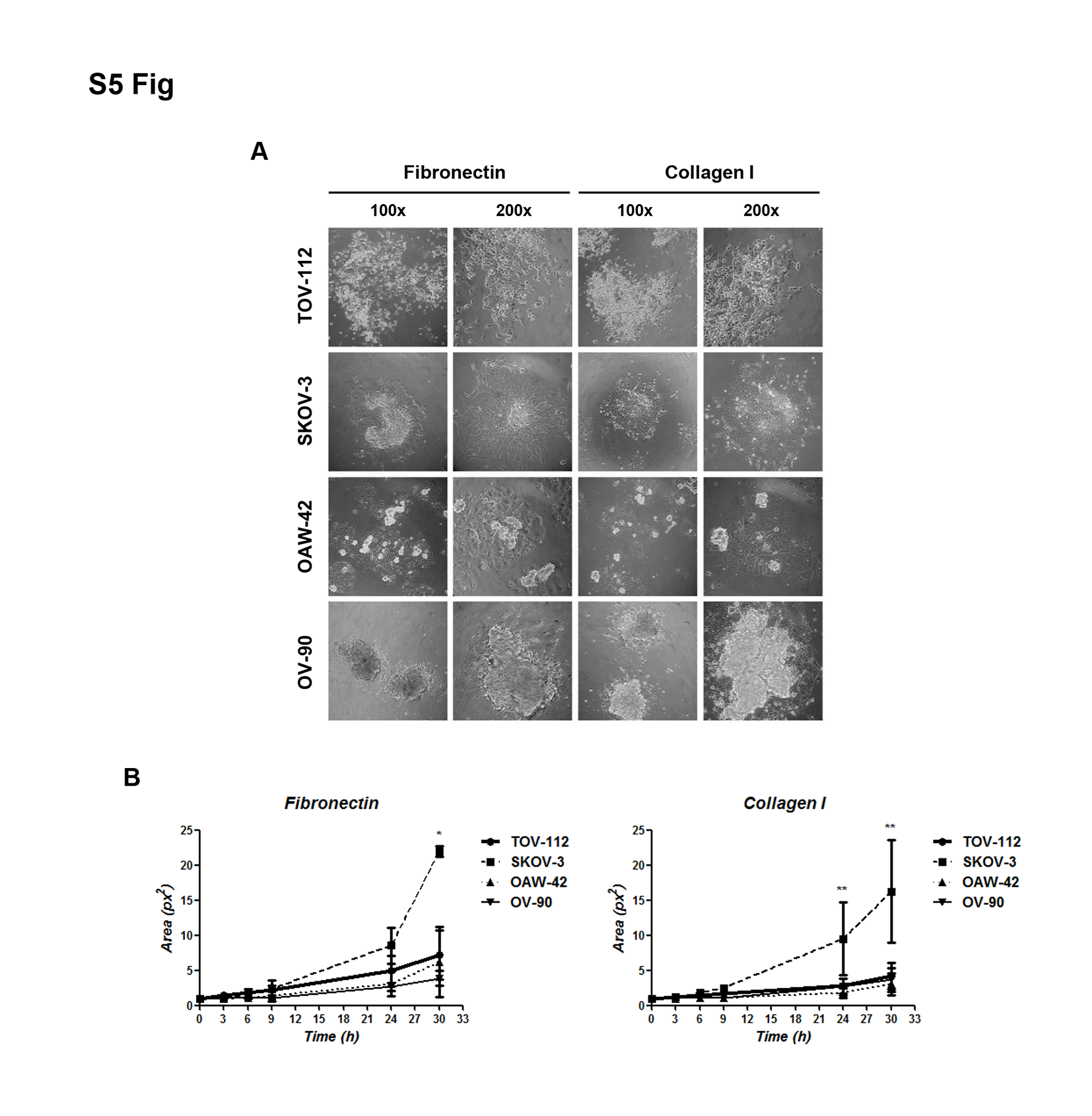

Supplement: S5 Fig — (A) Representative phase contrast images (100x and 200x magnifications) of TOV-112, SKOV-3, OAW-42 and OV-90 aggregates, disaggregating onto fibronectin and collagen I matrices after 30 hours. (B) Graphical representation of the area (px2: pixeles2) of TOV-112, SKOV-3, OAW-42 and OV-90 aggregates disaggregating onto fibronectin (left) and collagen I (right) as a function of time (h). (TIF) [file pone.0184439.s005.tif]
